# The impact of smoking and alcohol consumption on rosacea: a multivariable Mendelian randomization study

**DOI:** 10.3389/fpubh.2024.1320932

**Published:** 2024-02-19

**Authors:** Zhaowei Chu, Mengyao Yi, Cong Yan, Bingjie Li, Huan Zhang, Kun Guo, Songmei Geng

**Affiliations:** Department of Dermatology, The Second Affiliated Hospital of Xi'an Jiaotong University, Xi'an, China

**Keywords:** smoking, alcohol, rosacea, Mendelian randomization, causality

## Abstract

**Backgrounds:**

Observational studies have shown that cigarette smoking is inversely associated with risk of rosacea, However, it remains uncertain whether this association is causal or it is a result of reverse causation, and whether this association is affected by drinking behaviors.

**Methods:**

This study utilized the summary-level data from the largest genome-wide association study (GWAS) for smoking, alcohol consumption, and rosacea. The objective was to investigate the effect of genetically predicted exposures to smoking and alcohol consumption on the risk of developing rosacea. Two-sample bidirectional Mendelian randomization (MR) was applied, accompanied by sensitive analyses to validate the robustness of findings. Furthermore, multivariable MR was conducted to evaluate the direct impact of smoking on rosacea.

**Results:**

A decreased risk of rosacea was observed in individuals with genetically predicted lifetime smoking [odds ratio (OR)_MR − IVW_ = 0.53; 95% confidence interval (CI), 0.318–0.897; *P* = 0.017], and number of cigarettes per day (OR_MR − IVW_ = 0.55; 95% CI, 0.358–0.845; *P* = 0.006). However, no significant associations were found between initiation of regular smoking, smoking cessation, smoking initiation, alcohol consumption and rosacea. Reverse MR analysis did not show any associations between genetic liability toward rosacea and smoking or alcohol drinking. Importantly, the effect of lifetime smoking and the number of cigarettes per day on rosacea remained significant even after adjusting for alcohol consumption in multivariable MR analysis.

**Conclusion:**

Smoking was causally related to a lower risk of rosacea, while alcohol consumption does not appear to be associated with risk of rosacea.

## 1 Introduction

Rosacea is a common chronic inflammatory skin disease characterized by facial flushing, erythema, papules, pustules, telangiectasia, and rhinophyma (1). The visible “drunken” appearance and uncomfortable symptoms like burning and tingling significantly impact the quality of life for patients with this disease (2). Smoking and alcohol consumption have been extensively studied for their association with rosacea (3, 4). Epidemiologic studies have suggested an intriguing pattern, where cigarette smoking appear to be inversely associated with rosacea. However, inconsistent findings on this topic have led to debates regarding the nature of this association and its causality.

Multiple observational studies have suggested a potential link between smoking and a lower risk of rosacea. A case-control study in the U.K. reported that smoking was associated with a substantially reduced risk of developing rosacea (5). Furthermore, a recent meta-analysis demonstrated that current smoking was associated with a decreased risk of rosacea (6). However, a study conducted in Turkey presented contradictory findings, they reported a significantly higher risk of developing rosacea, particularly the erythema of subtype 1 rosacea, among smokers (7). Similarly, conflicting results also exist regarding the association between alcohol consumption and rosacea. Sunyun Li reported that increased alcohol intake was associated with a significantly elevated risk of rosacea (8), whereas other prospective cohort studies showed no association between drinking and rosacea (9). These discrepancies are likely attributed to the inherent limitations of observational studies, including the non-randomized design, residual confounding, and the possibility of reverse causation. For example, the above-mentioned meta-analysis on smoking and rosacea risk primarily used case-control studies, which may be susceptible to recall bias in the included data (6). Additionally, it is worth noting that the largest study included in the meta-analysis was conducted among patients on antihypertensives, which could potentially introduce bias in the assessment of rosacea cases. On the other hand, the study conducted in Turkey was a cross-sectional study (7), which inherently has limitations in establishing causality as it lacks a temporal dimension.

Recent advances in large-scale genome-wide association studies (GWASs) have identified specific genetic loci associated with smoking and alcohol consumption (10–12). Utilizing these genetic variants as instrumental variables, Mendelian randomization (MR) has emerged as a valuable method to strengthen causal inferences in the relationship between these exposures and outcomes (13). MR can be considered a “genetic randomized control trial,” as it randomly distributes genetic variants across the population during conception, mitigating biases from environmental confounding and reverse causality commonly observed in other observational methods (14).

In this study, we utilized two-sample Mendelian randomization analysis to investigate the potential relationship between genetically predicted smoking and rosacea, as well as the association between alcohol intake and rosacea. Additionally, we conducted a multiple variable MR analysis to investigate the direct effects of each exposure on the development of rosacea.

## 2 Methods

### 2.1 Data source

The largest GWAS summary data for smoking (cigarettes per day, initiation of regular smoking, smoking cessation, and smoking initiation) and alcohol intake (drinks per week) were from the GWAS and Sequencing Consortium of Alcohol and Nicotine use (GSCAN), it was a meta-analysis of over 30 GWAS on smoking and drinking in 1,232,091 participants with European ancestry (10), and the data were download from https://conservancy.umn.edu/handle/11299/201564. GWAS of lifetime smoking index was derived by Wootton et al. in 462,690 individuals of European ancestry from the UK Biobank (12), and the data was download from https://data.bris.ac.uk/data/dataset/10i96zb8gm0j81yz0q6ztei23d. A GWAS meta-analysis of alcohol consumption (log transformed g/day) with 480382 European individuals was conducted by Evangelou et al. (11), and the data was provided by the original paper. For rosacea, the genetic summary statistics finn-b-L12_ROSACEANAS was obtained from the FinnGenBiobank (https://r9.finngen.fi/), it consisted of 2,210 cases and 361,140 controls.

### 2.2 Genetic instrumental variables selection

**Figure 1:**
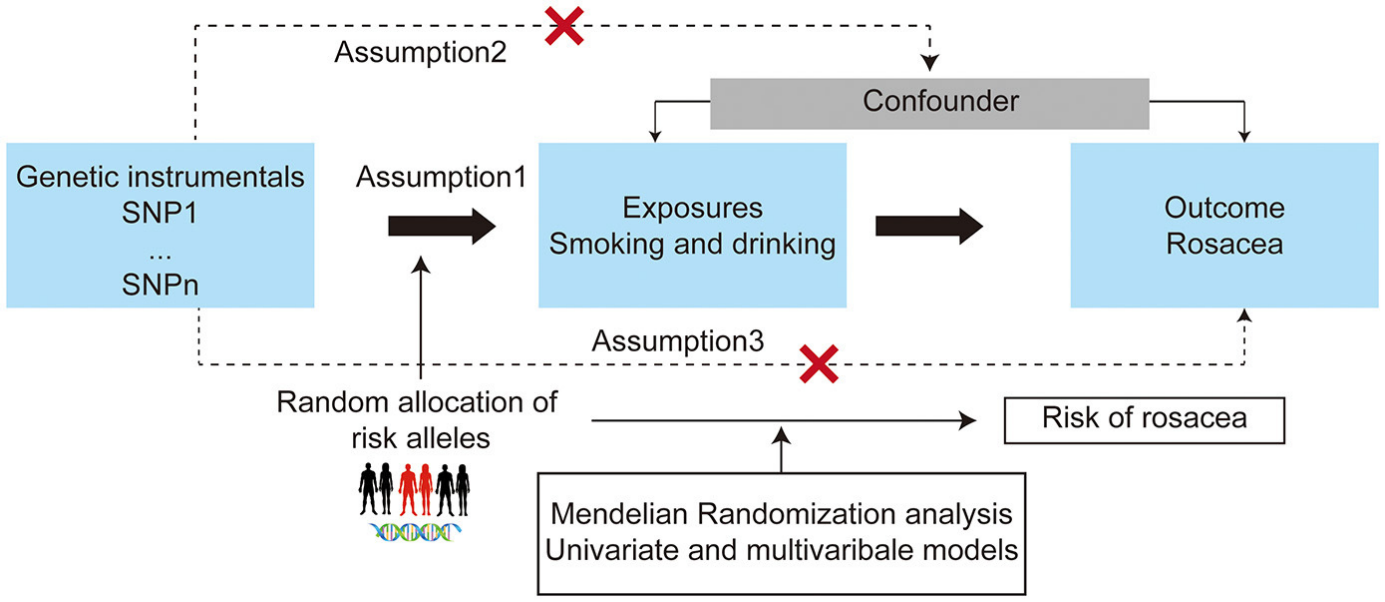
Study design overview and assumptions of the Mendelian randomization (MR) design. The dashed lines indicate possible causal effects between variables that may be against the MR assumptions. Assumption 1: the instrumental variables should be closely related to the risk factor of interest; assumption 2: the instrumental variables should not be associated with potential confounders, and assumption 3: the instrumental variables should affect the risk of outcome only through risk factors and not through other alternative pathways. SNPs, single nucleotide polymorphisms.

The MR approach based on the assumptions depicted in Figure 1 was used in this study (15). The single nucleotide polymorphisms (SNPs) associated with smoking (cigarettes per day, initiation of regular smoking, smoking cessation, and smoking initiation) and drinks per week at the genome-wide significance threshold (P < 5 × 10^−8^), as identified by the GSCAN study, were included in our analysis (10). Additionally, 126 SNPs identified as instrumental variables for the lifetime smoking index (12), and 46 novel SNPs associated with alcohol consumption at genome-wide significance (*P* < 5 × 10^−8^) from the GWAS meta-analysis were included (11, 12). After applying linkage disequilibrium clumping (*r*^2^ < 0.001, clump distance < 10,000 kb) and excluding SNPs strongly associated with rosacea, the remaining SNPs were utilized as instrumental variables for each condition. For rosacea, SNPs that reached the genome-wide significance threshold (*P* < 1 × 10^−6^) and demonstrated no linkage disequilibrium (defined as *r*^2^ < 0.001, clump distance < 10,000 kb) were selected as instrumental variables. In order to address potential confounding factors, each instrument SNP was thoroughly investigated in the PhenoScanner GWAS database (16), the SNPs with pleiotropic associations with potential confounders, such as obesity, inflammatory bowel disease, allergic disease, and other lifestyle traits, were excluded (Supplementary Table 1J) (17–19).

### 2.3 Univariate MR analysis

For each phonotype of smoking or drinking, the inverse variance weighted (IVW) method was used as the main MR analysis after harmonization of the direction of exposure and outcome. In addition to IVW, we also conducted sensitivity analyses using the weighted median and MR-Egger methods (20–22). Cochran Q value was calculated as a measure of heterogeneity among used instruments. The MR-Egger intercept analysis and MR-PRESSO approach were used to test horizontal pleiotropy and identify outliers (23). The F-statistic of each SNP was calculated to test instrument strength, with an *F*-value >10 indicating sufficiently strong.

### 2.4 Multivariable MR analysis

Considering that smoking was related to the alcohol intake, two-sample multivariable MR (MVMR) analysis was performed to estimate the direct effect of number of cigarettes per day and lifetime smoking index on rosacea conditioned on GSCAN GWAS of alcohol intake. For MVMR analyses, the instrumental SNPs were meeting the single-variable MR selection criteria described previously, and the multivariable MR extension of the IVW MR method was used to correct for measured pleiotropy (24). MR analysis was performed with R version 4.3.1 using the “TwoSampleMR”, “MVMR”, and “MRPRESSO” packages. The odds ratios (ORs) and 95% confidence intervals (CIs) were used to present the causal effects of exposure on outcome.

## 3 Results

### 3.1 Two-sample MR analysis for the causal association between smoking and rosacea

The SNPs selected as genetic instruments for all the examined conditions are listed in Supplementary Table 1.

**Figure 2:**
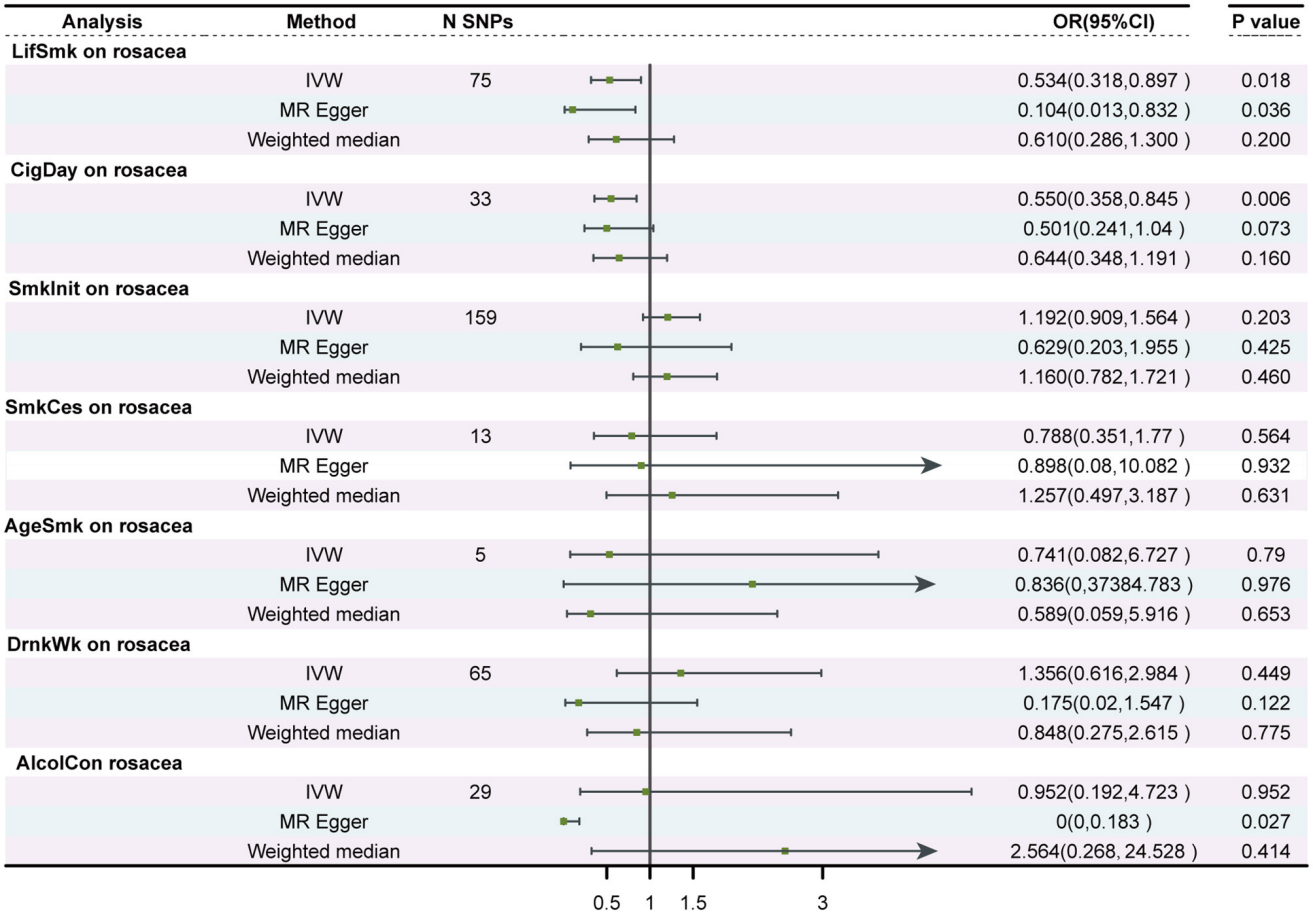
Forest plot to visualize the causal effect of smoking and alcohol consumption on rosacea. SNPs, single-nucleotide polymorphisms; OR, odds ratio; CI, confidence interval; LifSmk, lifetime smoke; CigDay, number of cigarettes per day; SmkInit, smoking initiation; AgeSmk, age at initiation of regular smoking; SmkCes, smoking cessation; DrnkWk, alcohol intake (drinks per week); AlcolCon, Alcohol consumption.

Using 75 SNPs that were robustly and independently associated with the lifetime smoking index, univariable MR analysis showed that lifetime smoking was associated with a decreased risk of rosacea [IVW Odds Ratio (OR), 0.53; 95% confidence interval (CI), 0.318–0.897; *P* = 0.017]. The sensitivity analysis with MR-Egger and weighted median method produced similar effect estimates with consistent direction (Table 1, Figure 2, and Supplementary Figure 1). The association between smoking and a decreased risk of rosacea was further supported by MR analysis using 33 SNPs associated with the number of cigarettes per day (IVW OR, 0.55; 95% CI, 0.358 to 0.845; *P* = 0.006) (Table 1, Figure 2, and Supplementary Figure 2). The association was consistent although it was not significant in the in the analyses by MR-Egger and weighted median method. However, we found no evidence of a causal relationship between age at initiation of regular smoking (IVW, *P* = 0.519), smoking cessation (IVW, *P* = 0.564), and smoking initiation (IVW, *P* = 0.087) on rosacea (Supplementary Figures 3–5).

**Table 1:** Univariate MR analysis of smoking/alcohol drinking and rosacea.

**Exposure/outcome**	**Methods**	**nSNP**	**Beta**	**SE**	**OR (95%CI)**	***P*-value**	**Test of heterogeneity**		**Test of pleiotropy**		
							***Q***	***P*** **-value**	**Egger-intercept**	**SE**	***P*** **-value**
LifSmk/rosacea	IVW	75	−0.628	0.265	0.534 (0.318, 0.897)	0.018	52.284	0.974	0.026	0.016	0.116
	MR-Egger	75	−2.265	1.062	0.104 (0.013, 0.832)	0.036	49.748	0.983			
	Weighted median	75	−0.494	0.386	0.61 (0.286, 1.3)	0.2					
CigDay/rosacea	IVW	33	−0.597	0.219	0.55 (0.358, 0.845)	0.006	24.751	0.816	0.003	0.010	0.757
	MR-Egger	33	−0.692	0.373	0.501 (0.241, 1.040)	0.073	24.653	0.783			
	Weighted median	33	−0.44	0.314	0.644 (0.348, 1.191)	0.16					
SmkInit/rosacea	IVW	159	0.176	0.138	1.192 (0.909, 1.564)	0.203	160.845	0.422	0.013	0.011	0.257
	MR-Egger	159	−0.463	0.578	0.629 (0.203, 1.955)	0.425	159.530	0.429			
	Weighted median	159	0.149	0.201	1.16 (0.782, 1.721)	0.46					
SmkCes/rosacea	IVW	13	−0.238	0.413	0.788 (0.351, 1.77)	0.564	23.964	0.021	−0.005	0.045	0.912
	MR-Egger	13	−0.108	1.234	0.898 (0.080, 10.082)	0.932	23.936	0.013			
	Weighted median	13	0.228	0.475	1.257 (0.495, 3.187)	0.631					
AgeSmk/rosacea	IVW	5	−0.299	1.125	0.741 (0.082, 6.727)	0.79	6.529	0.163	−0.002	0.106	0.983
	MR-Egger	5	−0.179	5.463	0.836 (0.37384.783)	0.976	6.528	0.089			
	Weighted median	5	−0.529	1.177	0.589 (0.059, 5.916)	0.653					
DrnkWk/rosacea	IVW	65	0.304	0.402	1.356 (0.616, 2.984)	0.449	75.024	0.163	0.026	0.013	0.053
	MR-Egger	65	−1.745	1.113	0.175 (0.02, 1.547)	0.122	70.626	0.237			
	Weighted median	65	−0.165	0.574	0.848 (0.275, 2.615)	0.775					
AlcolCon/rosacea	IVW	29	−0.049	0.817	0.952 (0.192, 4.723)	0.952	20.219	0.856	0.086	0.036	0.125
	MR-Egger	29	−10.408	4.443	0 (0, 0.183)	0.027	14.592	0.975			
	Weighted median	29	0.941	1.152	2.564 (0.268, 24.528)	0.414					

LifSmk, lifetime smoke; CigDay, number of cigarettes per day. SmkInit, smoking initiation; SmkCes, Smoking cessation; AgeSmk, age of initiation of regular smoking; DrnkWk, drinks per week; AlcolCom, Alcohol consumption. SNPs, single-nucleotide polymorphisms; OR, odds ratio; CI, confidence interval.

The *P*-value for the intercept in MR-Egger was larger than 0.05, indicating no horizontal pleiotropic effect. No SNP effect outliers were detected from MR-PRESSO and the Cochran Q-value indicated no heterogeneity.

### 3.2 Two-sample MR analysis for the causal association between alcohol consumption and rosacea

No evidence was found to support a causal relationship between genetically predicted alcohol consumption and rosacea, as assessed by alcohol intake (drinks per week) in the GSCAN GWAS (*P* = 0.449), or the novel SNPs associated with alcohol consumption identified by Evangelou E(*P* = 0.952) (Table 1, Figure 2, and Supplementary Figures 6, 7). No heterogeneity or horizontal pleiotropic effect was detected.

### 3.3 Reverse MR analysis for the causal association between rosacea and smoking and alcohol consumption

**Figure 3:**
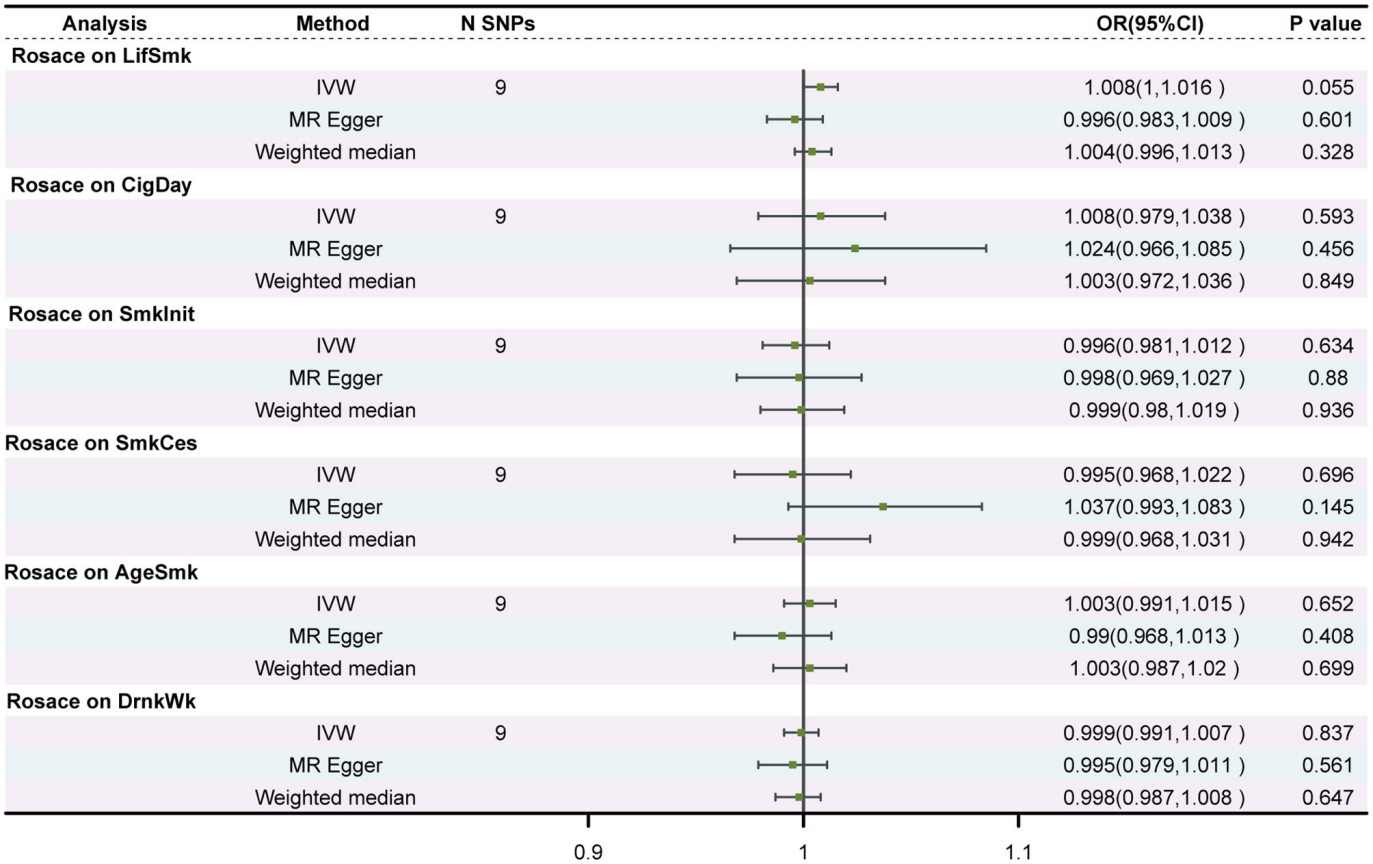
Associations of genetic predisposition to rosacea with smoking and drinking in two independent populations. SNPs, single-nucleotide polymorphisms; OR, odds ratio; CI, confidence interval; LifSmk, lifetime smoke; CigDay, number of cigarettes per day; SmkInit, smoking initiation; AgeSmk, age at initiation of regular smoking; SmkCes, smoking cessation; DrnkWk, alcohol intake (drinks per week); AlcolCon, Alcohol consumption.

For rosacea instruments, we selected SNPs reached the genome-wide significance threshold (P < 1 × 10^−6^). After linkage disequilibrium (*r*^2^ < 0.001, clump distance < 10,000 kb), we ended up with 9 SNPs that served as instruments for rosacea. The univariable MR analysis did not find evidence supporting reverse causality from rosacea to any smoking or drinking behaviors (Figure 3).

### 3.4 Multivariable MR analysis for direct causal effect of smoking on rosacea

In the multivariable MR analysis controlling for alcohol consumption, there was strong evidence for a direct reverse association between lifetime smoking and risk of rosacea (ORIVW, 0.495; 95% CI, 0.343 to 0.714; *P* = 1.68 × 10^−4^) (Table 2), as well as the reverse association between number of cigarettes per day and risk of rosacea (ORIVW,0.583;95% CI,0.348 to 0.976 *P* = 4.03 × 10^−2^). There was no evidence for heterogeneity or directional pleiotropy found. When controlling for lifetime smoking and number of cigarettes per day, there was still no evidence for a direct causal effect of alcohol consumption on risk of rosacea (*P* = 0.69, and *P* = 0.416, respectively).

**Table 2:** Multivariable MR analysis estimating the effect of lifetime smoking and cigarettes per day on rosacea, conditioning on Drink per week.

**Exposure**	**N SNPs**	**OR (95% CI)**	***P***
CigDay	33	0.495 (0.343, 0.714)	1.68 × 10^−4^
LifSmk	80	0.538 (0.348, 0.976)	4.03 × 10^−2^

SNPs, single-nucleotide polymorphisms; OR, odds ratio; CI, confidence interval; LifSmk, lifetime smoke; CigDay, number of cigarettes per day.

## 4 Discussion

Observational studies have shown that current smokers have a ~34% decreased risk of developing rosacea (5). However, there are debates on this association. Some argue that reverse causation may play a role, as patients with rosacea maybe more likely to quit smoking during the presence of disease (5). Others argue that the association observed in long-term ex-smokers does not support this hypothesis (6).

In this study, we conducted both univariable and multivariable Mendelian Randomization and discovered that smoking was causally associated with a lower risk of developing rosacea. The effect of smoking on rosacea were consistent, whether considering lifetime smoking on rosacea risk or the number of cigarettes smoked per day on rosacea risk. However, no causal association was found between alcohol consumption and the risk of developing rosacea. The results were largely robust to sensitivity analyses accounting for horizontal pleiotropy.

Our finding on smoking and alcohol drinking in relation to rosacea is in line with most previous studies. A prospective study involving 95 809 US women in 2017, as well as another cohort study involving 59,973 Taiwanese, both suggested that current smoking is associated with a decreased risk of rosacea (25, 26). These results are consistent with a comprehensive systematic analysis of 12 published articles, which also found a reduced risk of rosacea in current smokers (6). Furthermore, it is worth noting that the general trend of smoking prevalence is significantly higher among men than women, while the proportion of men with rosacea is half lower than compared to women (27). This intriguing observation could potentially indicate an inverse association between smoking and rosacea. However, it is important to acknowledge that some other observational studies have reported conflicting results, like a prospective cross-sectional study showing increased rosacea risk among smokers (7). Furthermore, previous research has shown distinct effects of past and current smoking on rosacea risk, with current smoking reducing the risk and former smoking increasing it (6). This discrepancy can be attributed to variations in study design, demographics, exposure duration, patient inclusion/exclusion criteria, regional influences, and limitations of conventional observational research for identifying causal effects.

Although the precise mechanisms remain to be elucidated, multiple biological mechanisms are hypothesized to mediate the potential beneficial role of smoking in rosacea development. Firstly, vasodilation caused by neurovascular dysfunction has been implicated in the pathogenesis of rosacea. Cigarette smoking has been shown to cause vasoconstriction in peripheral arteries, which may counteract the vasodilation associated with rosacea (26). Additionally, a high prevalence of contact allergies has been found in rosacea patients, suggesting a role of allergic reactions in its development (19). Cigarette smoke can mitigate allergies by diminishing the reaction of immune cells, particularly the activity of mast cells, to allergens (28). Furthermore, as previously mentioned, rosacea is now well-established as a chronic inflammatory disorder. Nicotine, a prominent component of tobacco, exhibits robust anti-inflammatory properties (29), thereby potentially reducing the risk of developing rosacea.

This MR study revealed a non-genetic association between alcohol consumption and rosacea using the largest GWAS data to date. The finding aligns with a recent meta-analysis of 14 studies, which concluded that alcohol consumption is not a significant overall risk factor for rosacea (30). However, we also noticed previous studies indicating that increased alcohol intake is linked to a higher risk of rosacea in US women (8), and subgroup analysis of the aforementioned meta-analysis demonstrated that alcohol consumption increases the risk of phymatous rosacea (30). These results suggest that the relationship between alcohol consumption and rosacea may vary depending on gender and subtypes of rosacea. Further research is necessary to investigate this association within specific subgroup populations as GWAS data becomes available.

The major strength of this study is MR design, which overcome potential bias from confounding and reverse causality (14). Admittedly, the results of this study should be interpreted with caution, considering both its limitations and those typical of MR. Firstly, although we carefully selected highly associated SNPs, they only partially account for smoking and drinking behaviors. Secondly, the MR approach assesses cumulative lifelong genetic effects, and should not be extrapolated to presume the effect of a time-limited behavior change. Thirdly, GWAS data for smoking and drinking rely partly on self-reported information, potentially susceptible to recall bias. Fourthly, our analysis focused on Europeans, limiting the generalizability of our findings to other populations. However, future research can address this limitation by incorporating GWAS data from diverse regions and ethnicities. Finally, the study's statistical power is relatively low owing to the small sample size of the outcome GWAS (Supplementary Table 1K). Future research should also consider conducting subgroup analyses based on sex and rosacea severity when specific GWAS data for these populations become accessible.

In summary, this study utilized the largest available genetic datasets for exposure and outcome to perform MR analysis and investigate the causal relationship between smoking, alcohol consumption, and rosacea. Our findings indicate that genetically predicted exposures of lifetime smoking and number of cigarettes per day was causally related to lower risk of rosacea, while no genetic evidence supporting a causal association between alcohol consumption and rosacea was found. Importantly, the effect of smoking on rosacea risk was independent of alcohol consumption. Further research is warranted to investigate the underlying mechanisms driving this association.

## 5 Conclusions

Our findings indicated that genetically predicted smoking behavior including cigarettes per day and life time smoke was associated with a significant lower risk of rosacea. No significant associations were found between alcohol consumption and rosacea. The association between smoking and a lower risk of rosacea was not affected by alcohol consumption. This association provides clues for understanding pathogenesis of rosacea, and developing novel therapeutic interventions for rosacea. However, given the extensive health risks associated with cigarette smoking, it remains crucial to advocate promote smoking cessation efforts.

## Data availability statement

The original contributions presented in the study are included in the article/Supplementary material, further inquiries can be directed to the corresponding authors.

## Author contributions

ZC: Conceptualization, Formal analysis, Methodology, Software, Writing—original draft, Writing—review & editing. MY: Methodology, Software, Writing—original draft. CY: Methodology, Software, Validation, Visualization, Writing—original draft. BL: Methodology, Validation, Writing—review & editing. HZ: Writing—review & editing. KG: Supervision, Validation, Writing—review & editing. SG: Funding acquisition, Supervision, Writing—review & editing.
