# Supplementary material for: The impact of smoking and alcohol consumption on rosacea: a multivariable Mendelian randomization study
Source: Front Public Health. 2024 Feb 19;12:1320932. doi: 10.3389/fpubh.2024.1320932 (PMC10909955; doi:10.3389/fpubh.2024.1320932)
Supplement: Supplementary file 1 [file Data_Sheet_1.docx]

Supplementary figure

Supplementary fig 1.


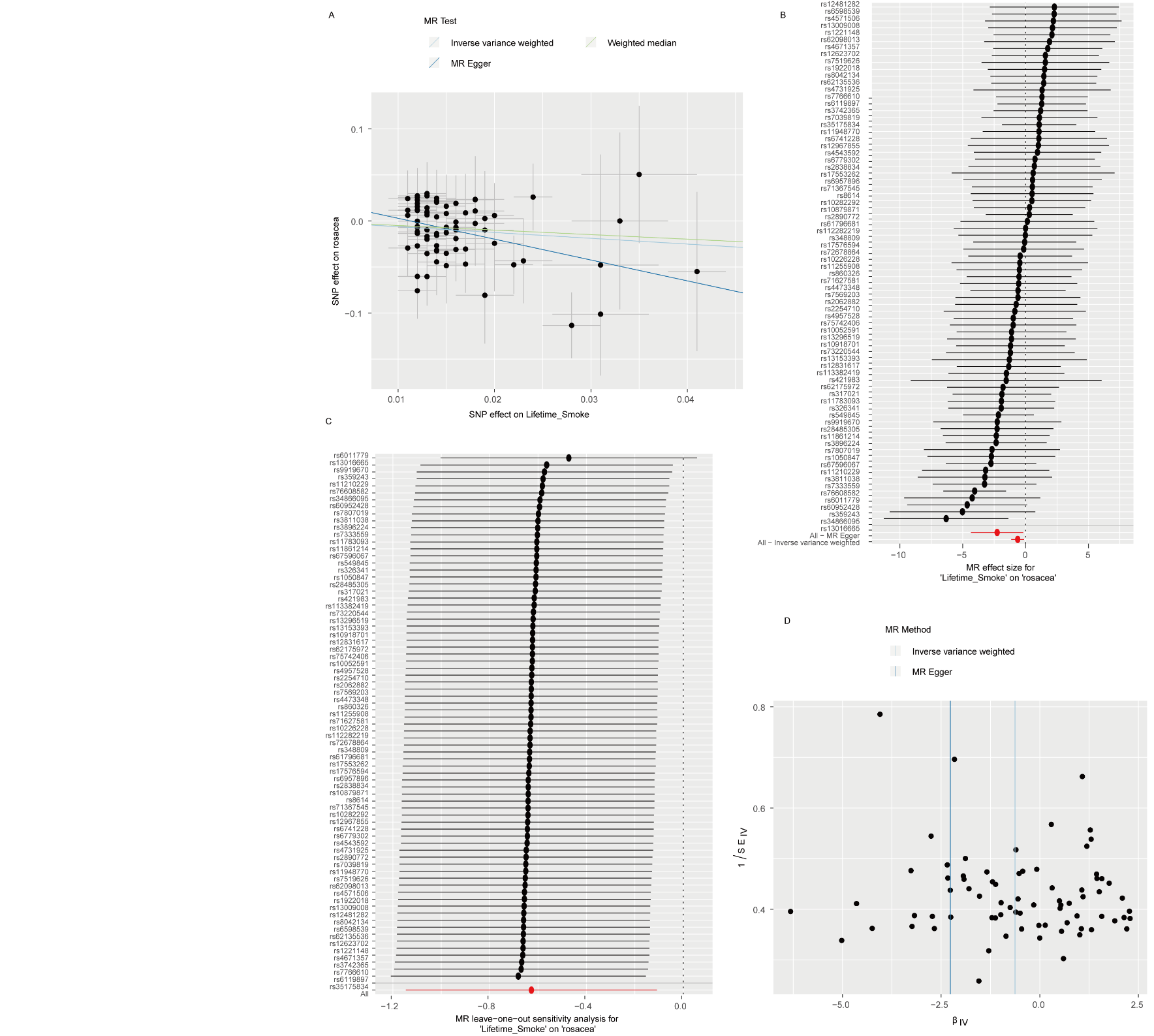


Supplementary fig 1. Forest plot(A), leave-one-out sensitivity analysis(B), scatter plot(C), and funnel plot (D)of the effect of lifetime smoking index (LifSmk) on rosacea.

Supplementary fig 2.


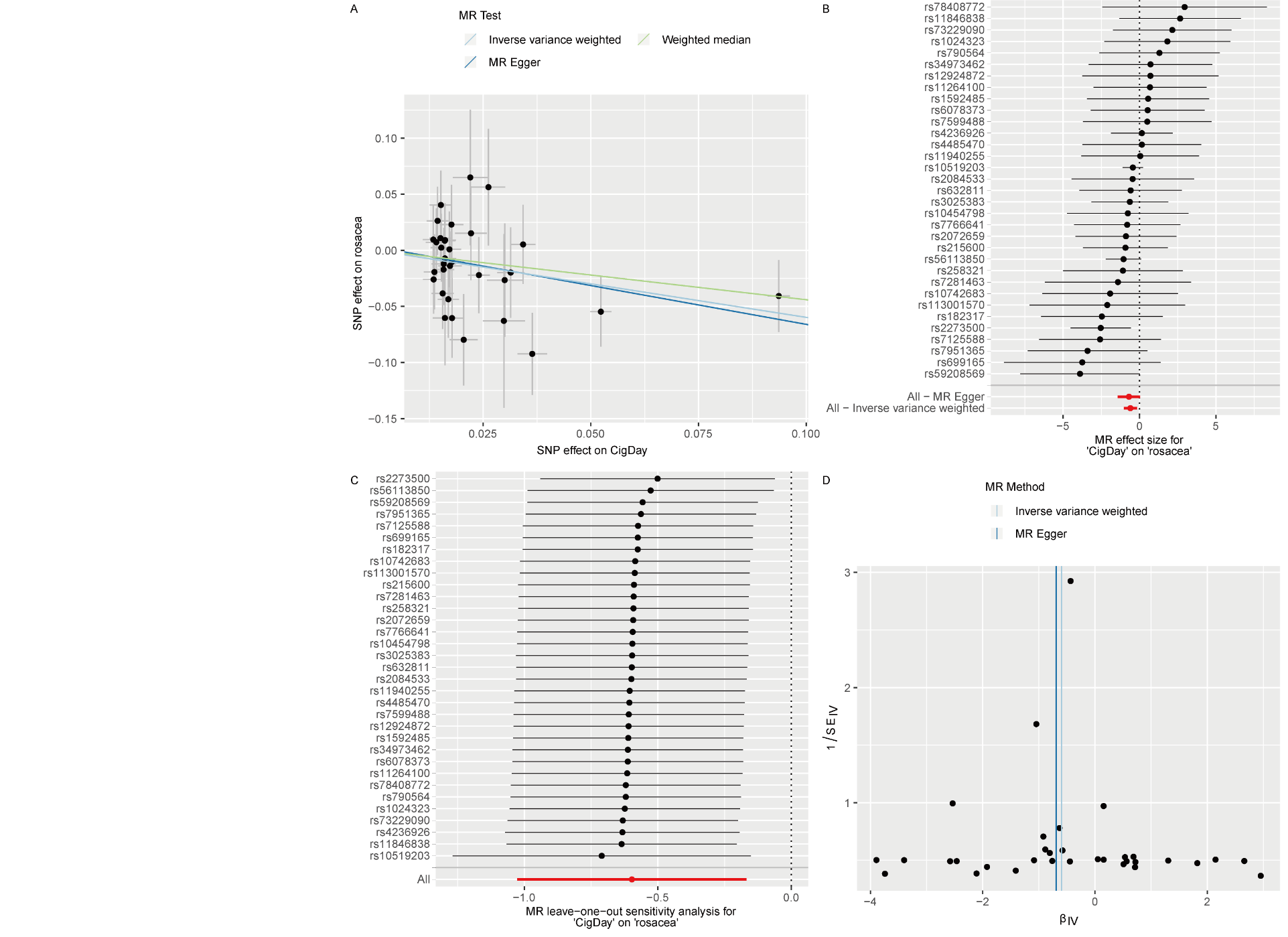


Supplementary fig 2. Forest plot(A), leave-one-out sensitivity analysis(B), scatter plot(C), and funnel plot (D)of the effect of cigarettes per day (CigDay) on rosacea.

Supplementary fig 3


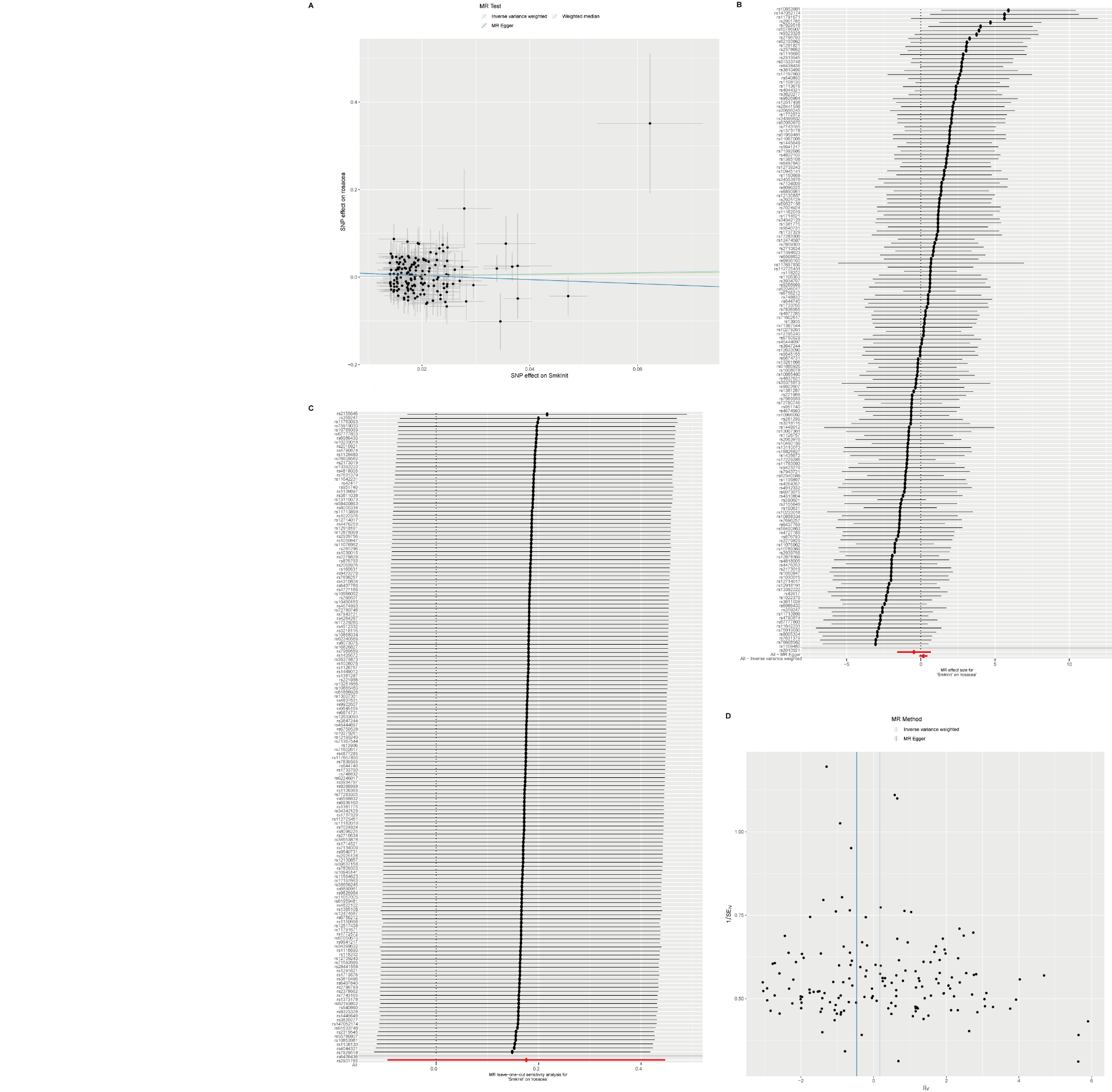


Supplementary fig 3. Forest plot(A), leave-one-out sensitivity analysis(B), scatter plot(C), and funnel plot (D)of the effect of smoking initiation (SmkInit) on rosacea.

Supplementary fig 4


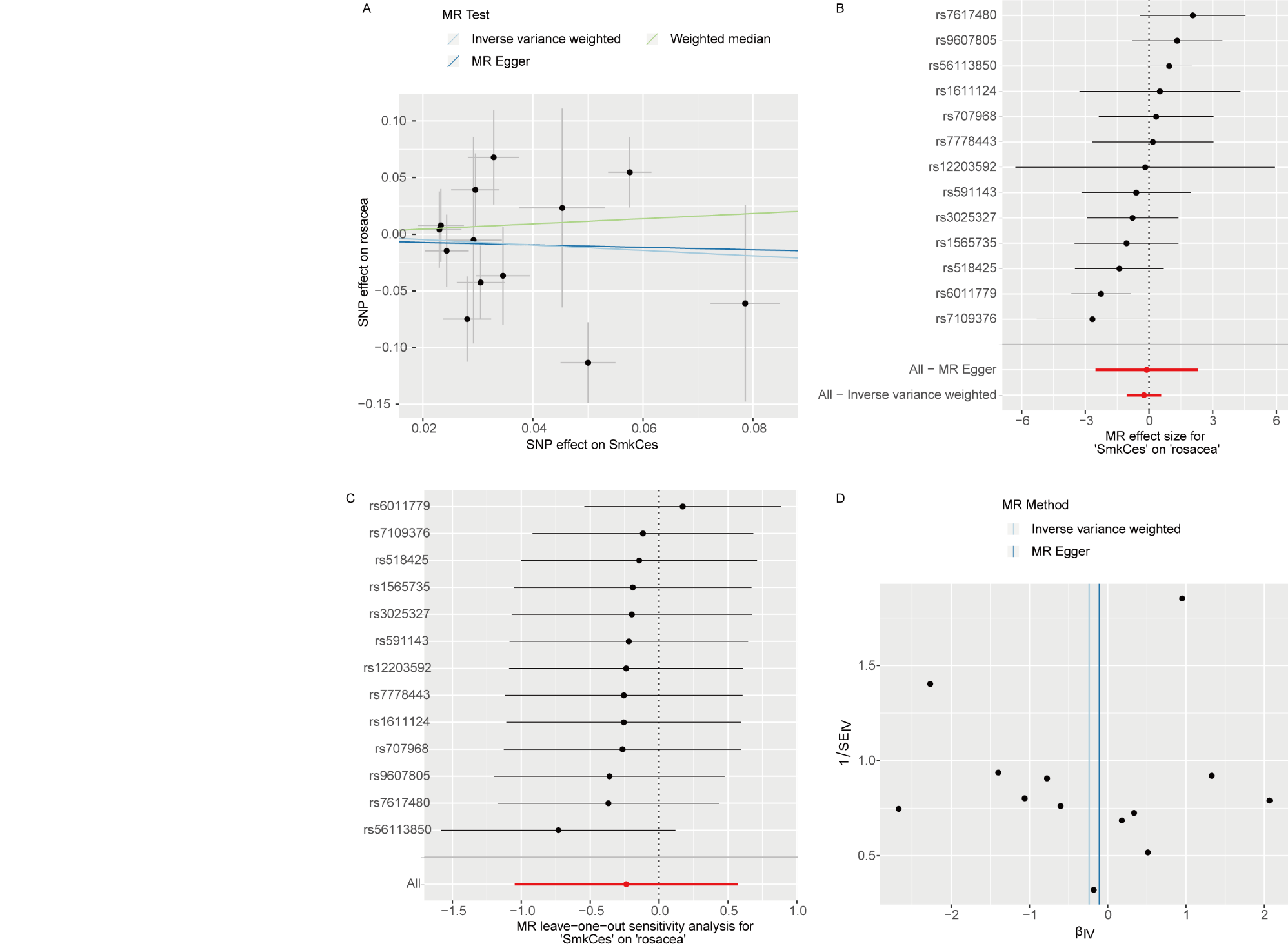


Supplementary fig 4. Forest plot(A), leave-one-out sensitivity analysis(B), scatter plot(C), and funnel plot (D)of the effect of smoking cessation on rosacea.

Supplementary fig 5.


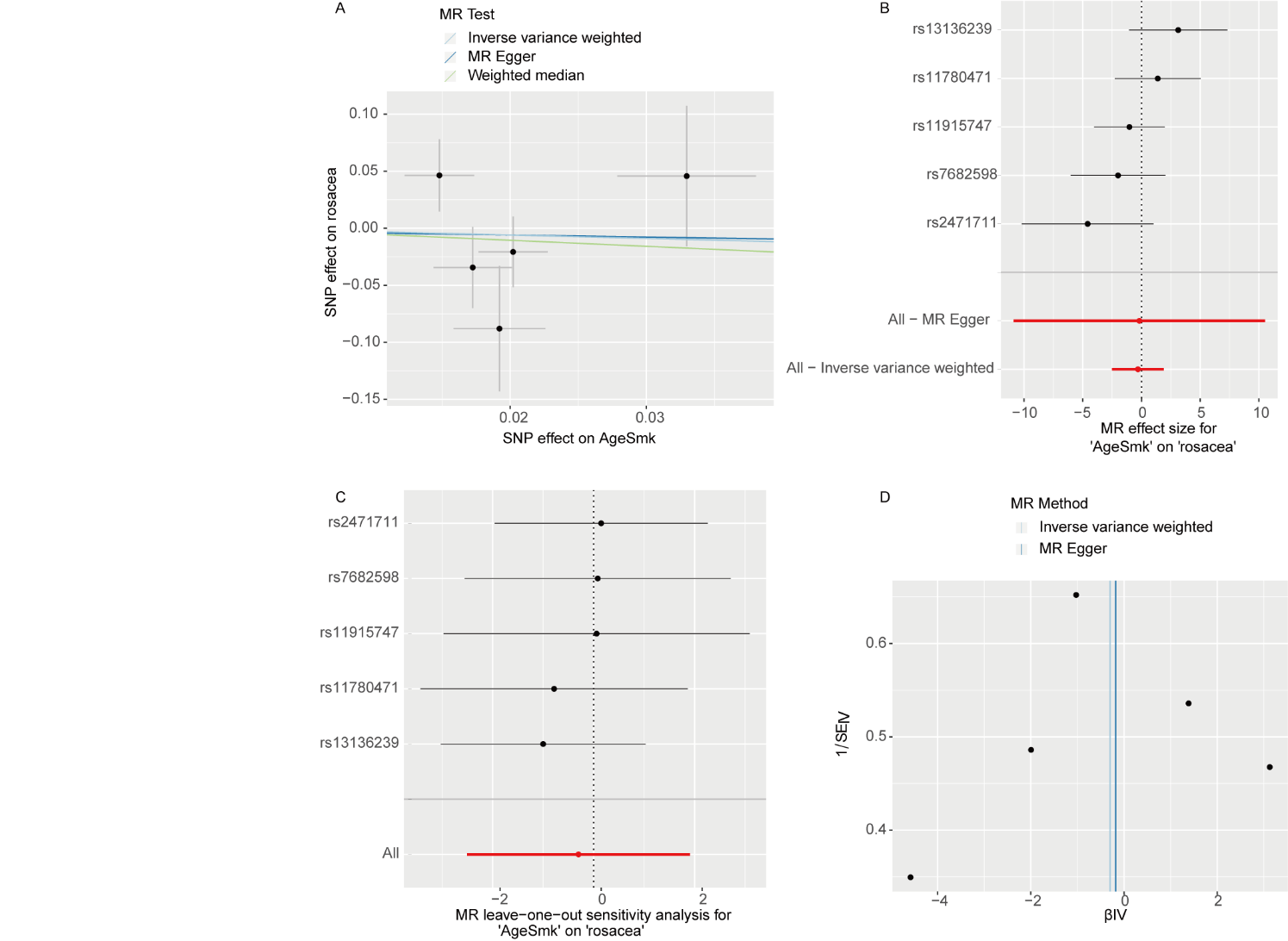


Supplementary fig 5. Forest plot(A), leave-one-out sensitivity analysis(B), scatter plot(C), and funnel plot (D)of the effect of age of initiation of regular smoking (AgeSmk) on rosacea.

Supplementary fig 6


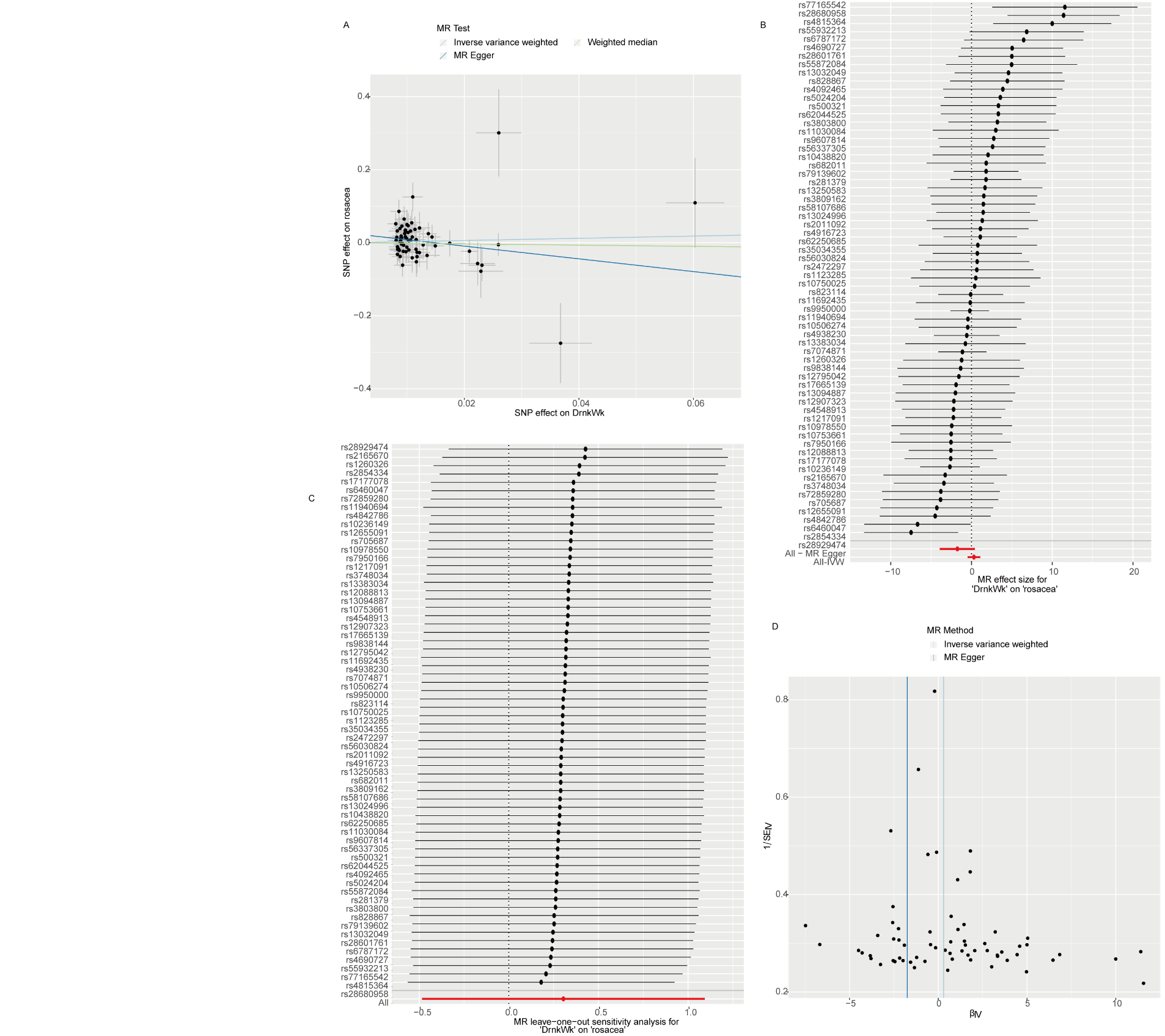


Supplementary fig 6. Forest plot(A), leave-one-out sensitivity analysis(B), scatter plot(C), and funnel plot (D)of the effect of drinks per week (DrnkWk) on rosacea.

Supplementary fig 7


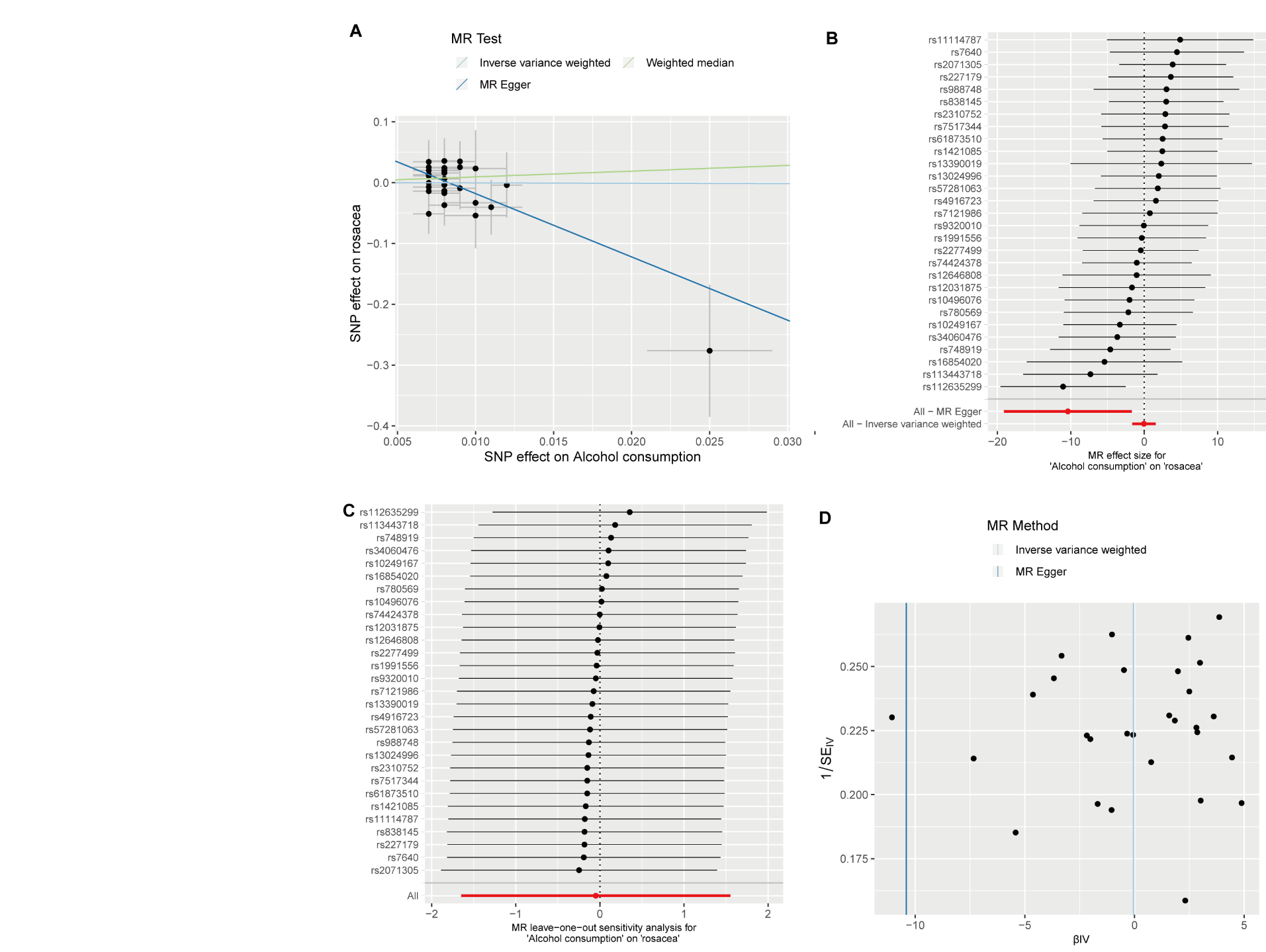


Supplementary fig 7. Forest plot(A), leave-one-out sensitivity analysis(B), scatter plot(C), and funnel plot (D)of the effect of alcohol consumption (AlcolCon) on rosacea.
